# Oral complications associated with metal ion release from oral piercings: a systematic review

**DOI:** 10.1007/s40368-023-00831-0

**Published:** 2023-08-15

**Authors:** M. Masood, L. J. Walsh, S. Zafar

**Affiliations:** School of Dentistry, The University of Queensland, 288 Herston Road, Herston Qld, Brisbane, QLD 4006 Australia

**Keywords:** Oral, Piercings, Metal ions, Complications

## Abstract

**Purpose:**

This systematic review explored dental complications associated with metal ion release from oral piercings using the Preferred Reporting Items for Systematic Reviews and Meta-analyses guidelines.

**Methods:**

Item retrieval from October 2022 to December 2022 from databases, such as Cochrane Central Register of Controlled Trials, Medline, PubMed, Embase, Scopus and Web of Science, using predefined search terms was undertaken by two independent reviewers. Data were extracted and risk of bias was assessed using the Joanna Briggs Institute (JBI) critical appraisal checklist. From 1509 identified studies, 25 were included for analysis.

**Results:**

Of the 25 studies, 20 included both clinical examination and questionnaire-based data. The remaining five studies were deemed low quality based on the Joanna Briggs Institute criteria. The average time piercings were worn ranged between 5 and 48 months. Most studies did not investigate complications from metal ion release. Only two studies examined the direct effects of metal ion release and showed that metal ions may cause hypersensitivity reactions and mucosal changes. Other soft tissue complications were reported, with gingival recession noted in 16 out of 25 studies, especially due to lip piercings. Studies reporting other complications were as follows: swelling (9), pain (8), infection (6), bleeding (6), inflammation (5), alterations to speech, eating and/or swallowing (5), changes to taste or metallic taste (5), and mucosal changes (4). Ten studies reported tooth chipping from tongue piercings.

**Conclusion:**

Oral piercings leach metal ions into surrounding tissues which may cause local mucosal changes. Furthermore, oral piercings cause damage to both soft and hard oral tissues, particularly gingival recession for lip piercings and tooth chipping for tongue piercings. Thus, to prevent such adverse injuries, dental professionals should discourage patients against oral piercings.

**Supplementary Information:**

The online version contains supplementary material available at 10.1007/s40368-023-00831-0.

## Introduction

Oral piercings are a form of body jewellery that are inserted into the oral or peri-oral tissues (Ziebolz et al. 2009). They have a long history, having been practised in diverse ancient cultures as part of ceremonial and religious practices (Vozza et al. 2014; Hennequin-Hoenderdos et al. 2016; Chadwick et al. 2005; Covello et al. 2020). However, over the last two decades, oral piercing has evolved into a common practice amongst young people in the Western world (Ziebolz et al. 2009; Vozza et al. 2014; Chadwick et al. 2005) with approximately 5.2% of young adults having oral piercings (Hennequin-Hoenderdos et al. 2016). Oral piercings are placed into the tongue, lips, cheeks or frena (Chadwick et al. 2005; Levin et al. 2005). Tongue piercings are commonly a barbell, with balls at either end of a rod, whilst lip piercings may be a ring or a labret, which is a rod with one ball and one flat disc (Ziebolz et al. 2009; Chadwick et al. 2005; Levin et al. 2005).

Common short-term complications after placing an oral piercing include pain, swelling of the site, and secondary bleeding (Ziebolz et al. 2009; Chadwick et al. 2005; Levin et al. 2005; Inchingolo et al. 2011). These problems are often self-limiting and of short duration. Erythema and haematoma can also occur, whilst tongue oedema may cause airway obstruction (Ziebolz et al. 2009; Levin et al. 2005). Changes to speech, swallowing patterns and mastication have also been reported (Kapferer et al. 2010; Firoozmand et al. 2009; Vieira et al. 2011). Less frequently, the generation of a galvanic current flow between barbell tongue piercings and metal dental restorations has been noted (Chadwick et al. 2005). Long-term complications from oral piercings include damage to gingival tissues, fractures of enamel and dentine, and allergic reactions, especially to nickel (Ziebolz et al. 2009; Hennequin-Hoenderdos et al. 2016; Levin et al. 2005). Abrasion of the enamel, fractures of tooth structure, and gingival recession, particularly affect the mandibular anterior teeth, are common long-term complications (Kapferer et al. 2010; Firoozmand et al. 2009; Kapferer et al. 2012; Vilchez-Perez et al. 2009).

The majority of piercings are made from metal, including surgical-grade stainless steel, titanium, platinum, silver or gold (Ziebolz et al. 2009; Walsh et al. 2008; Masood et al. 2023). When oral piercings come in contact with oral fluids such as saliva, they may corrode and leach out metal ions into the surrounding tissues (Domingo et al. 2019). The released ions can then cause allergic reactions, with nickel reported as the most common allergen (Vozza et al. 2014; Levin et al. 2005; Lupi et al. 2010). Other metals, such as chromium and cobalt, have been also reported to cause hypersensitivity reactions (Domingo et al. 2019; Lupi et al. 2010). Surface defects on the piercing surface may be a location where corrosion begins (Domingo et al. 2019). In addition to causing local reactions in the oral mucosa, released ions may enter the systemic circulation. Systemic complications of oral piercings include bacteraemia and infections at distant sites, including infective endocarditis and brain abscesses (Domingo et al. 2019). Whether other systemic reactions, including toxicity from released heavy metals, occurs from oral piercings is unclear.

Despite a range of serious complications caused by leaching of metal ions from oral piercings, no reviews of the literature on this topic have been conducted. A recent systematic review examined only the complications of oral piercings on hard and soft tissues (Passos et al. 2022). Therefore, the aim of this review is to investigate the oral complications as a result of metal ions released from oral piercings. The null hypothesis for this review is that the oral complications will be associated with the release of the metal ions from oral piercings.

## Materials and methods

### Protocol and registration

This systematic review used The Preferred Reporting Items for Systematic Reviews and Meta-analysis (PRISMA) guidelines (Page et al. 2021). The protocol for this review was registered on PROSPERO (CRD42022336112).

### Eligibility criteria, screening and selection

The eligibility for inclusion was based on the PICO criteria as described in Table 1 with (P) human participants; (I) intervention or oral metal piercings used; (C) comparison: allergy or hypersensitivity reactions and/or ion release from oral piercings; (O) outcome: incidence of hard and soft tissue injuries/complications from oral piercings and incidence of dental complications from metal ion release from oral piercings. The PICO research question for this systematic review was “Is there an association between metal ions released from oral piercings and oral complications?”

**Table 1:** Inclusion and exclusion criteria adopted in the literature search

Inclusion criteria	Exclusion criteria
P: Participants: human subjects of any gender, mean age below 25 years	Studies on piercings in other body areas (e.g. ear)
I: Interventions: Oral or peri-oral metal piercings located on tongue and/or lip and/or frenum and/or cheeks	Papers in alterative languages to English with no English translation
C: Comparison: allergy or biological or hypersensitivity reaction in relation to oral piercings; ion release from oral piercings	
O: Outcomes: Incidence of hard and soft tissue injuries/complications from oral piercings. Adverse material reactions/hypersensitivity Incidence of dental complications from metal ion release from oral piercings	Reviews, abstracts, letters to the editor, editorials
Study design: Nonrandomised control trials, cohort studies and case–control studies	
Publication year: no restrictions Language: papers in English or with English-language abstract available	

### Search strategy

Cochrane Central Register of Controlled Trials (CENTRAL), Medline (via EbscoHost), PUBMED (National Library of Medicine), Embase (via Elsevier), Scopus and Web of Science databases were searched using combinations of the following key terms: (mouth* OR oral OR lip OR tongue OR labial OR buccal OR frenulum OR mucosa OR gingiva AND (piercing* OR body piercing OR jewellery (disease OR injury OR complications) AND (allergy OR toxicity OR adverse reaction OR hypersensitivity) AND (release of metal ions OR metal ion release OR metal ions). The search was conducted from October 2022 to December 2022. Additional hand searches of article reference lists as well as Google and grey literature searches were conducted using the same keywords to identify literature not accessible via standard databases, as given in the supplemental data table.

### Data collection and analysis

Studies reporting complications from oral or perioral piercings were identified. After screening of titles and abstracts, the reference lists of applicable studies were examined to identify any additional sources. The studies were imported into Endnote™ software (Clarivate, U.S.A), and duplicate articles were removed. The first level of selection was undertaken using titles and abstracts, by applying the inclusion and exclusion criteria outlined in Table 1. Subsequently, full text versions were retrieved, and a second level of selection was performed, using the same criteria. Study characteristics included in the data collection forms were as follows: author/s, year published, country where research was undertaken, study design, total number of subjects, participant characteristics (gender, mean age, type of piercing, mean period piercing worn), post-piercing oral complications, whether metal ion release was reported, conclusions and key findings. These findings were tabulated using Excel spreadsheets. For synthesis, studies were grouped into whether metal ion release was reported or not. Inter-rater reliability between the two independent assessors (M.M and S.Z) during the data extraction stages (identification, screening, eligibility, and inclusion) was calculated as a percentage. Inter-rater reliability agreement for selection and outcome was evaluated using Cohen’s kappa using the Statistical Package for the Social Sciences version 24.0 software (IBM Corp., Armonk, NY, USA).

### Risk bias assessment reporting

The Joanna Briggs Institute (JBI) critical appraisal checklist for non-randomised experimental studies was used to rate the studies in terms of their risk of bias (Porritt et al. 2014). Two independent reviewers (M.S and S.Z) screened the articles, and any discrepancies were resolved by discuss with the third author (L.W).

## Results

### Literature results

From the database searches, a total of 1506 articles were identified, whilst three additional articles were found via hand-searching and checking reference lists. During the first level of selection, 254 duplicates were removed, and 1155 records excluded due to irrelevant titles and/or abstracts. A total of 100 papers were screened for eligibility. Case reports and series were excluded (*n* = 53) and articles with participants of mean age above 25 years of age were excluded (*n* = 17). This resulted in 30 articles that met the second level of inclusion criteria for this review.

From the 30 articles selected for inclusion, 5 articles were excluded when applying the selection criteria and through discussion amongst the reviewers. Three articles included only English abstracts. For these, it was difficult to assess study methodology against the JBI criteria, and so they were excluded. Two articles were not included due to being irrelevant, with one discussing Candida infections and another looking solely at temporomandibular disorders, which is outside the inclusion criteria. This reduced the final number of studies to 25.

**Fig. 1:**
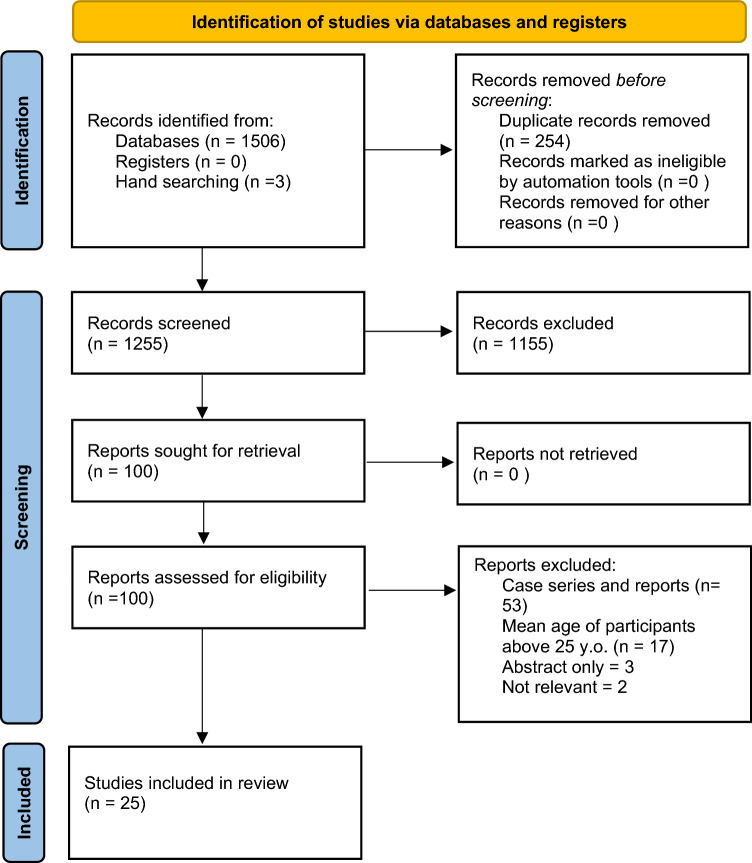
PRISMA (Preferred Reporting Items for Systematic Reviews and Meta-analysis) flowchart for selection of studies

The Preferred Reporting Items for Systematic Reviews and Meta-analysis (PRISMA) flowchart is shown in Fig. 1. No papers reported an objectionable risk of bias.

### Inter-rater reliability agreement

The inter-rater reliability percentages for the two assessors were as follows: identification 98.8% (1509/1527 studies), screening 98.4% (1255/1276 studies), eligibility 100% (100/100 studies) and inclusion 83.3% (25/30 studies). It was agreed that two studies discussed the outcome of complications related to metal ion release from oral piercings. Discrepancies were resolved via discussion and agreement reached. The kappa scores were as follows: for inclusion 76.09% agreement (kappa 0.516) and for outcome 96.15% agreement (kappa 0.8999).

### Outcomes from included studies

The included studies consisted of 14 cohort, 6 case–control and 5 cross-sectional studies. Three studies were published between 2020 and 2015, 12 studies between 2014 and 2009 and 10 studies, 2008 and 2003.

From the 25 included studies, the greatest number of articles published was from Brazil (*n* = 4). Other locations studies for studies are shown in Table 2.

**Table 2:** Characteristics of included studies

Author year Country	Study design	Gender Mean Age (SD) Range in years	Type of piercing Mean period for wearing piercing	Post piercing dental/oral complications	Outcome of metal ion release	Conclusions/Key findings
Samoilenko et al. [2019] Ukraine	Cohort	*N* = 36, F only Age: 18–32 yrs	Total piercings: 37 Tongue: *n* = 22; Lip: *n* = 9 AWP: 24.11 ± 0.7 months	Complications (total: *n* = 37) Pain 58.3%; Oedema 47.2%; Bleeding 11.1%; Dental fractures 35.1%; Gingival recession 29.7%; Mucosal atrophy 21.6%; Tooth sensitivity 19.4%; Plaque on piercing 43.2%; Longer barbell stem length cause greater recession and chipping	Not reported	Oral piercings were Associated damage to hard tissues, gingival recession, and soft tissue atrophy
Domingo et al. [2019] Argentina	Cohort	*N* = 16, (F: 10, M: 6); Age: 11–21 yrs	Total piercings: *n* = 11 Lower lip *n* = 8; Upper lip *n* = 6; Tongue *n* = 4 AWP range: 5–48 months	Lichenoid lesions on cheek and lip mucosa (*n* = 4); Elevated greyish lesions associated with metallosis on lip (*n* = 4); Mobility upper right molar and tearing at the piercing site (*n* = 4)	Cytological smears and EDS analysis showed: Aluminium (66.6%), Tungsten (5.5%), Molybdenum (2.7%)	Ion particles are released from metal piercings and may have caused the observed lesions
Tomazevic et al. [2017] Slovenia	Case Control	Total: 45 *Pierced group*: n = 17 (F:15; M:2; Age: 22 yrs); *Control group*: n = 28 (F:22; M:6; Age: 22.4 yrs)	Tongue piercing: *n* = 17 AWP: 31.8 months	Complications from piercings (*n* = 17) Total Dental injuries: 76.5%; Enamel infractions: 23.5%; Enamel fracture: 58.8%; Enamel/dentine fracture: 5.9%	Not reported	Subjects with tongue piercing have higher risks of dental injury including enamel fractures
Simoes et al. [2014] Brazil	Cross sectional	Total:82 (F:63; M: 19; Age: 14–30 yrs)	Total piercings: *n* = 109 Lip: *n* = 55; Tongue: *n* = 28; Lingual frenum: *n* = 5; Labial frenum: *n* = 18; Cheek: *n* = 3 AWP: < 6 months: 24.8%; 6–12 months: 13.8%; > 12 to 24 months: 11.9%; > 24 months: 49.5%	Gingival recession 39.4%; Changed sensation: 16%; Altered saliva: 13%; Dental fractures 11.9%; Lip depressions 9.2%; Mucosa depressions 2.6%; Inflammation 2.8%; Labial frenum oedema 1.8%; Keloid scar 1.8%; Tongue depression 0.9%; Lip hyperplasia 0.9%; Tooth mobility 0.9%	Not reported	Oral piercings were associated with complications involving hard and soft dental tissues with the most frequent complication being gingival recession
Kapferer et al. [2012] Austria	Cross sectional	Total: 47 (F: 34; M: 13; Age: 16–24 yrs)	Lower lip labret piercing adjacent to first premolar: *n* = 5; Lower lip labret piercing adjacent to mandibular canine: *n* = 42 AWP: 37.3 ± 33.6months	Mid-buccal recession (*n* = 4) with canine and first premolars most affected; Tooth chipping (*n* = 1); Plaque levels significantly higher in test than control; Hyperplastic tissue (4.3%) around the stud closure (*n* = 2)	Not reported	Lateral lower lip piercings were associated with significantly higher rates of buccal recession, tooth chipping and plaque accumulation
Plessas et al. [2012] Greece	Cohort	Total:110 (F: 58; M: 52; Age: 18–35 yrs)	Total: 161 pierced sites Tongue: *n* = 51; Lip: *n* = 110 AWP: 30.3 months	Self-reported complications (*n* = 110 subjects): Inflammation (*n* = 53); Gingival recession (*n* = 21); Dentine hypersensitivity (*n* = 21); Increased salivary flow (*n* = 15); Taste change (*n* = 11); Dental defects (*n* = 52); Soft tissue ulcer (*n* = 88); Tissue overgrowth (*n* = 12); Gingival recession (*n* = 64); GR defects with Miller Class II (*n* = 10); Biting piercing and caused dental defects (*n* = 43); Striking piercing and caused dental defects (*n* = 12)	Not reported	Tongue and lip piercings caused increased dental defects, gingival recession, attachment loss and increased probing depths of teeth adjacent to pierced sites
Ziebolz et al. (2012) Germany	Case control	Total: 92 (M only) Age: 21–22 yrs *Test group*: n = 46 *Control group*: n = 46	Tongue piercing: *n* = 46 AWP: 45.6 ± 36 months	Complications (total = 46): Enamel cracks (*n* = 38); Enamel defects (*n* = 29); Gingival lingual recession 59% (*n* = 27)	Not reported	Tongue piercing showed increased occurrence of enamel defects, enamel cracks and lingual recessions
Vieira et al. [2011] Brazil	Cohort	Total: 39 (F: 26; M: 13; Age: 18–24 yrs)	Total piercings: *n* = 42 Tongue: *n* = 37; Upper lip: *n* = 1; Lower lip: *n* = 4 AWP: 15 months	Complications (total = 42) Excessive bleeding (*n* = 29); Pain (*n* = 22); Syncope (*n* = 2); Swelling (*n* = 26); Parafunctional habits (*n* = 35); Tongue lacerations (*n* = 13); Palate traumas (*n* = 8); Gingival recessions (*n* = 2); Tooth fracture (*n* = 1)	Not reported	Oral piercings caused mainly local complications related to soft and hard dental tissues
Hickey et al. [2010] France	Cohort	Total:201 (F: 146; M: 55; Age: 22.7 yrs)	Tongue piercing *n* = 106; Lip piercing *n* = 55; Cheek piercing *n* = 7 AWP: 19.2 months	Gingival recession 8.5%; Chipped teeth 6.9%; Tooth chipping 21.4%; Infection 3%; Swelling 51.7%; Sensitivity 1.5%; Taste disturbance 12.3%; Speaking discomfort 67%; Eating difficulty 78.3%; Difficulty swallowing 28.4%; Increased saliva 20.4%	Not reported	The occurrence of complications from oral piercings is high, and public education or awareness is needed
Kapferer et al. [2010] Austria	Cohort	Total:189 (F: 153; M: 36; Age: 13–36 yrs)	Total: *n* = 210 Lip piercings: *n* = 130 Tongue piercings: *n* = 80 AWP:43.5 ± 33.1 months	*Early Complications:* Mild pain (*n* = 124); Swelling (*n* = 70); Mild infection (*n* = 35); Speaking problem (*n* = 48); Problems eating (*n* = 99) *Late Complications:* Recurrent infection (*n* = 13); Gingiva recession (*n* = 45); Tooth chipping (*n* = 13); Tooth movement (*n* = 4)	Not reported	Pain was the most common early complications. Late complications included gingival recession, tooth chipping and recurrent infections
Lupi et al. [2010] Italy	Case control	Total: 30 *Pierced tongue: (*F: 12; M: 3; Age: 20–29 yrs) *Control: (*F: 12, M: 3; Age: 20–29 yrs)	Tongue piercings: *n* = 15 AWP: 46.1 months	Mucosal smears surrounding piercing body showed a copious and diffuse bacterial flora (*n* = 10); Fungi hyphae and spores located intercellularly (*n* = 3); Neutrophil infiltration (*n* = 5); Cytolysis of epithelial cells (*n* = 4)	Light and SEM: showed foreign metallic bodies in keratinocytes indicated ion release from piercings	Ion release from oral piercings may be related to direct toxic effects and delayed reactions of metal sensitisation
Oberholzer et al. [2010] Australia	Cohort	Total:250 (F: 195; M 55; Age range: 16–35 yrs)	Tongue piercing Frenum piercings AWP: not stated	Total subjects with piercings: *n* = 250 Tooth damage/chipping (*n* = 85); Gum problems (*n* = 40); Infections (*n* = 20); Soft tissue damage (*n* = 149)	Not reported	Damage to hard dental tooth structure was the most common complication
Pires et al. [2010] Brazil	Case control	Total:180; Age = 5–21 yrs) *Control group:* 120 (F: 77; M: 43; *Tongue piercing:* 60 (F 33; M 27)	Tongue piercing: *n* = 60 AWP: 24 months (range: 25 months ± 2.84 months)	Swelling (*n* = 11); Infection (*n* = 4); Inflammation (*n* = 4); Keloid formation (*n* = 1); Dental fracture (*n* = 12); Habit of biting piercing (*n* = 30); Tongue piercing have 11 times greater chance of gingival recession	Not reported	Tongue piercings were strongly associated gingival recession in the anterior lingual mandibular region compared to control
Firoozmand et al. [2009] Brazil	Cohort	*N* = 33; (F: 15; M: 18; Age: 14–18 yrs)	Tongue piercings: *n* = 22; Lower lip: *n* = 10; Other: *n* = 3 AWP: NS	Swallowing difficulty 2.6%; Fractured teeth 5.10%; Local inflammation 7.70%; Gingival recession 10.25%; Keloid formation 10.25%; Bacterial plaque 17.90%; Tongue fissure 20.50%	Not reported	Oral piercings were associated with local complications to gingiva and teeth
Vilchez-Perez et al. [2009] Spain	Cross sectional	*N* = 50 (F: 39; M:11; Age: 18–24 yrs)	Total lip piercings: *n* = 76 Labret type: *n* = 58; Ring: *n* = 18 AWP: 35.4 months ± 19.5	Gingival recession (*n* = 17); Tooth fractures and cracks (*n* = 15); Mucosal alterations (*n* = 7); Hyperplastic tissues near piercing (*n* = 5); Greatest gingival recession around canine and first bicuspid teeth	Not reported	Lower lip piercings enhanced gingival recession and were associated with tooth fractures and cracks
Ebrahim and Naidoo [2008] South Africa	Cohort	*N* = 126 (F: 107; M: 19; Age: 14–24 yrs)	Tongue piercing: (*n* = 110); Lip piercing: (*n* = 16); Both tongue and lip:(*n* = 10) AWP: 37% piercing for > 2 yrs; 23% between 1 and 2 yrs; 17% for 6–12 months; 23% < 6 months	Pain 69.05%; Swelling 52.38%; Difficulty eating, speaking and swallowing 70.63%; Irritation 33%; Taste changes 19%; Itchiness 10%; Chipped teeth 10.31% (*n* = 10); Gingival recession (*n* = 2)	Not reported	Tongue and/or lip piercings were associated with trauma to teeth and gums
Slutzkey et al. [2008] Israel	Cohort	*N* = 303 (F: 177 M: 126; Age: 18–22 yrs)	Tongue and lip piercing: NS; AWP: NS	Gingival recession (*n* = 9)	Not reported	Gingival recession amongst young adults was related to oral piercings
Kapferer et al. 2007 Austria	Case control	*N* = 100; Age: 18–21 yrs Test: F: 50; M: 44; Control: F: 50; M = 6)	Lower lip labret studs: *n* = 50 AWP: 39.4 ± 3.5 months	Gingival recession 68%; Localised periodontitis 4%	Not reported	The prevalence of gingival recessions was associated with labial piercing
López-Jornet, et al. [2006] Spain	Cohort study	*N* = 97 (F: 68; M: 29; Age: 13–35 yrs)	Tongue: *n* = 45; Lip: *n* = 52; Cheek: *n* = 1 AWP: 14.07 months	Gingival recession (*n* = 23); Tooth damage (*n* = 13); Hypertrophy/atrophy (*n* = 9); Pain (*n* = 37); Swelling (*n* = 46); Bleeding (*n* = 8); Salivary flow (*n* = 14); Halitosis: (*n* = 4); Taste changes (*n* = 9)	Not reported	Labial piercing frequently caused gingival recession and damage to the periodontium
López-Jornet, et al. [2006] Spain	Cross sectional	*N* = 59 (F:48; M:11; Age = 13–25 yrs)	Tongue: *n* = 17; Lip: *n* = 13 AWP: 0–3 months: 21%; 3 months to 1 year: 54%; > 1 year: 25%	Pain (*n* = 3); Bleeding 24%; Gingival inflammation (*n* = 22), Gingival swelling (*n* = 8); Dental fractures or fissures/cracks (*n* = 6); Increased salivation (*n* = 6); Bad breath (*n* = 4); Metallic taste (*n* = 5)	Not reported	Tongue piercing was associated with pain, inflammatory reactions, and dental problems
Dougherty et al. [2005] USA	Case control	*N* = 46; Mean age:23.9 yrs Control: F: 14; M:9 Test: F:14; M: 9	Tongue piercings *n* = 23 AWP = 40.32 years,	Mandibular anterior teeth mobility (*n* = 2); Gingival recession (*n* = 9); Periodontal pocketing (*n* = 4); Broken teeth (*n* = 8); Radiographic calculus (*n* = 5)	Not reported	Wearing a tongue stud increases the risk of developing abnormalities in alveolar bone surrounding mandibular anterior teeth
Kieser et al. [2005] New Zealand	Cohort	*N* = 43 (F: 40; M: 3 Age: 14–34 yrs)	Tongue piercing: *n* = 33 Lip piercing: *n* = 15 Both tongue and lip: *n* = 5	Swelling/infection (*n* = 12); Pain (*n* = 6); Lymphadenopathy (*n* = 1); Abnormal tooth wear (*n* = 12); Gingival recession -Labial sites (*n* = 12); Lingual sites (*n* = 12)	Not reported	Oral piercings were associated with pain, swelling, localised gingival recession, and abnormal tooth wear
Levin et al. [2005] Israel	Cohort	*N* = 389 (F: 179; M: 210; Age: 18–24 yrs)	Tongue: *n *= 39; Lip piercing: *n* = 8; Other: *n* = 32 AWP: 13.04 months (range 1–60 months	After piercing complications: (total *n* = 79) Swelling (*n* = 41); Bleeding (*n* = 36); Fractured teeth (*n* = 11); Infection (*n* = 9); Gingival recessions (*n* = 21)	Not reported	Swelling, bleeding, tooth fractures and gingival recessions were common complications
Stead et al [2006] UK	Cohort	*N* = 145 Age:13–43 yrs	Tongue piercing (*n* = 123) AWP:29 months	Swelling (*n* = 120); Pain (*n* = 87), Difficulty eating (*n* = 81); Speech problems (*n* = 63); Bleeding (*n* = 53); Ingestion of jewellery (*n* = 42); Tooth fracture (*n* = 38); Plaque on jewellery (*n* = 37); enlarged piercing hole (*n* = 19)	Not reported	Tongue piercing may cause complications including swelling, pain, tooth damage and difficulty speaking and eating
Campbell et al. [2002] USA	Cross sectional	*N* = 52 (F: 21; M: 31; Age: 18–40 yrs)	Tongue piercings: *n* = 52 AWP: 20 ± 24 months	Mandibular central incisor lingual recession 50% (*n* = 26) Tooth chipping 47% in tongue piercing for 4 + years (*n* = 24)	Not reported	Tongue piercing was associated with lingual recession of mandibular anterior teeth and chipping of posterior teeth

*F* females, *M* males; *AWP* Average worn period, *NS* not specified

The majority of the studies included had more female participants than males, and only three studies had more male participants than females (Levin et al. 2005; Firoozmand et al. 2009; Campbell et al. 2002). One study examined only male participants (Ziebolz et al. 2012) and one study did not specify the gender distribution of subjects (Stead et al. 2006). In terms of the mean age of participants, the most common age was 22 years old (*n* = 6) and the youngest mean age was 14 years old (Domingo et al. 2019).

There were 7 studies that examined tongue piercings only, whilst 3 examined lip piercings only, and 15 studies examined both tongue and lip piercings (Table 2). Five studies examined piercings in other oral locations such as the cheek or frenum in addition to tongue and lip piercings. The average period that piercings were worn ranged between 5 and 48 months.

In terms of post-piercing oral complications, reported soft tissue problems included damage to gingival tissues, and mucosal changes, such as atrophy, hyperplasia or local lichenoid reactions. Hard tissue complications were tooth-related, such as fracture or chipping. Gingival recession was the most common soft tissue complication reported in 16 out of 25 studies. Tooth fracture or chipping was noted in 10 studies. Other commonly reported complications included: swelling (*n* = 9), pain (*n* = 8), infection (*n* = 6), bleeding (*n* = 6) and inflammation (*n* = 5). Alterations to speech, eating and/or swallowing were noted in five studies. Changes to taste, or a metallic taste were reported in five studies. Four studies reported mucosal changes or atrophy from oral piercings.

Most studies did not report complications resulting from metal ion release. Only two studies examined the direct effects of metal ion release on surrounding oral tissues (Domingo et al. 2019; Lupi et al. 2010). One study explored different piercing materials, such as titanium, stainless steel and Teflon, in terms of the rate of gingival recession and tooth chipping from these various types of oral piercings (Hickey et al. 2010).

When applying the JBI critical appraisal criteria for non-randomised experimental studies, 20 studies included both clinical examination and questionnaire-based data, and were rated as having good methodological quality (Levin et al. 2005; Firoozmand et al. 2009; Vieira et al. 2011; Kapferer et al. 2012; Vilchez-Perez et al. 2009; Domingo et al. 2019; Lupi et al. 2010; Campbell et al. 2002; Ziebolz et al. 2012; Dougherty et al. 2005; Kapferer et al. 2007; Kieser et al. 2005; Lopez-Jornet and Camacho-Alonso 2006; Lopez-Jornet et al. 2006; Oberholzer et al. 2010; Pires et al. 2010; Plessas et al. 2012; Samoilenko et al. 2019; Slutzkey et al. 2008; Tomaževič et al. 2017). Five studies were rated as having low quality methodology (Kapferer et al. 2010; Simoes et al. 2014; Stead et al. 2006; Hickey et al. 2010; Ebrahim et al. 2008). Four of these studies used only self-reported questionnaire data and no clinical examinations were undertaken (Kapferer et al. 2010; Stead et al. 2006; Hickey et al. 2010; Ebrahim et al. 2008). Reported risk bias for the included studies is provided in the supplemental data.

## Discussion

This systematic review aimed to examine the incidence of hard (teeth) and soft tissue complications relating to oral piercings, with a particular focus on complications caused by metal ion release. To the author’s knowledge, this is the first systematic review that examines metal ion release from oral piercings and the subsequent complications. The results of this review showed that metal ion release can occur from oral piercings when subject to the oral environment. They may corrode and release metal ions into surrounding oral tissues, causing local soft tissue reactions. Oral piercings are made from a variety of metals, such as surgical stainless steel, titanium, platinum, or gold, as mentioned above (Masood et al. 2023). Two included studies examining metal ion release investigated piercings made from surgical stainless steel only (Domingo et al. 2019; Lupi et al. 2010). Stainless steel piercings were found to cause a high incidence of tooth chipping (Hickey et al. 2010). One study that examined different oral piercing materials showed that titanium piercings cause greater gingival recession (Hickey et al. 2010). Teflon or acrylic piercings showed lower levels of dental complications, and the change in composition avoided the problem of metal allergy (Hickey et al. 2010; Dougherty et al. 2005).

Complications caused by metal ion release were reported by two studies (Domingo et al. 2019; Lupi et al. 2010); Domingo et al. (2019) examined both lip and tongue piercings, and reported the presence of metal particles, such as aluminium, tungsten and molybdenum, in keratinocytes at sites where the oral mucosa was pierced. Lupi et al. (2010) investigated only tongue piercings, and noted concentrations of iron, nickel, and chromium in tongue mucosal keratinocytes. These metal ions may exert local cytotoxic effects on keratinocytes (Lupi et al. 2010), which may result in local mucosal changes, such as atrophic, “hyperplastic, leukoedematous, erythematous, and/or erosive lesions” (Domingo et al. 2019). Interestingly, the rate of metal ion release is also influenced the surface quality of the piercings, where surface defects can be a site of metal corrosion (Masood et al. 2023; Domingo et al. 2019); Domingo et al. (2019) noted surface defects on both used and unused piercings.

Of the 25 articles included for analysis, there were no articles published prior to 2002 that examined lip or tongue piercings and related dental complications. The majority of the included studies had more female participants than males, indicated a higher prevalence of oral piercings in females (Walsh et al. 2008; Dougherty et al. 2005; Kapferer et al. 2007; Samoilenko et al. 2019; Simoes et al. 2014; Tomaževič et al. 2017). There may also be a higher willingness of females to participate in studies, and they may seek oral health input more readily than males (Dougherty et al. 2005; Kapferer et al. 2007).

The included studies generally reported that tongue piercings cause significantly more dental complications than lip piercings (Ebrahim et al. 2008). Pain, difficulty speaking and eating problems were reported more often with tongue piercings (Kapferer et al. 2010; Firoozmand et al. 2009; Vieira et al. 2011). These were similar findings to the 2022 systematic review by Passos et al. Immediately after piercing placement, common complications included pain, swelling, bleeding and irritation (Kapferer et al. 2007; Samoilenko et al. 2019; Ebrahim et al. 2008). One study showed that 86% of subjects with oral piercings experienced these complications (Kapferer et al. 2007). Pain was reported to occur in between 58.3 and 69% of cases and swelling in between 47.2 and 52% of cases (Kapferer et al. 2007; Samoilenko et al. 2019). Furthermore, mucosal changes were reported in 6 of the 25 studies included for analysis. Atrophy or de-papillation around the oral piercing was the most common mucosal change with tongue piercings (Oberholzer et al. 2010). Hyperplasia or tissue overgrowth at the pierced site was seen mainly in tongue piercings, at a prevalence of 16.2% to 33.3% (Vieira et al. 2011; Samoilenko et al. 2019). Complete resolution was achieved when the piercings were removed (Vieira et al. 2011).

Most studies showed that tongue piercings are associated with tooth injuries such as chipping or fractures (Firoozmand et al. 2009; Ziebolz et al. 2012; Oberholzer et al. 2010). The study by Tomaževič et al. (2017) showed that dental damage of any type, such as enamel fractures, enamel–dentin fractures, and complicated crown–root fracture, occurred twice as frequently in people with tongue piercings (76.5%) compared to community controls (32.1%). About 59% of people with tongue piercings had enamel fractures (Tomaževič et al. 2017). The majority of teeth with enamel damage (92%) were molars and premolars (Campbell et al. 2002). This may be explained by the habit of patients playing with, biting, or knocking the piercing against their teeth (Campbell et al. 2002). Of subjects with fractured teeth, this habit of playing with the piercing was noted in some 51.6% of cases (Hickey et al. 2010).

Lip or labial piercings cause more gingival recession (Kapferer et al. 2010; Firoozmand et al. 2009; Kapferer et al. 2012; Vilchez-Perez et al. 2009; Plessas et al. 2012). As such, labial piercings have been shown to be more damaging to periodontal tissues (Kieser et al. 2005; Lopez-Jornet and Camacho-Alonso 2006; Slutzkey et al. 2008). In contrast, Pires et al. (2010) noted that subjects with tongue piercings are at 11 times increased risk of developing lingual anterior gingival recession. This is supported by Dougherty et al*.* (2005) who reported that tongue piercings were commonly associated with the presence of calculus, which may lead to higher levels of periodontal disease. The systematic review and meta-analysis by Passos et al. showed that 33% of individuals with oral piercings had gingival recession. The problem of gingival recession is primarily related to the position of the piercing. Studies examining this issue showed that piercings placed at the CEJ caused a greater rate of buccal gingival recessions, (Kapferer et al. 2007) whilst lip piercings placed coronally to the CEJ had a lower incidence of lower lip buccal recession (Kapferer et al. 2012). This important finding regarding piercing placement is something which body piercing professionals should consider when advising patients about possible long-term complications.

The rate of oral complications may be related to piercing length and the wear period. The barbell rod or stem length on tongue piercings appears to be directly proportional to the risks of gingival recession and tooth chipping (Campbell et al. 2002). Longer stems cause more recession due to the greater ease of the barbell piercing coming into direct contact with the gingival tissues (Campbell et al. 2002; Hickey et al. 2010). Similarly, several studies also reported a higher prevalence of gingival recession and tooth chipping with longer wear periods (Campbell et al. 2002; Plessas et al. 2012; Ebrahim et al. 2008). Interestingly, one study found that the greatest incidence of complications occurred within 2 years and involved 57.6% of cases (Lopez-Jornet and Camacho-Alonso 2006).

Multiple studies reported limitations to their study design. The main limitation reported was convenience sampling, where the participants were recruited specifically for participation in the study (Stead et al. 2006; Dougherty et al. 2005; Kieser et al. 2005; Ebrahim et al. 2008). Some studies did not have a control group (Domingo et al. 2019; Plessas et al. 2012), which prevents comparisons with the experimental group. Lastly, the majority of studies included a questionnaire in the study design, with four studies relying solely on the information collected from the survey to draw conclusions (Kapferer et al. 2010; Stead et al. 2006; Hickey et al. 2010; Ebrahim et al. 2008). Memory bias is a key limitation that may influence the results from questionnaire-based studies (Stead et al. 2006; Kapferer et al. 2007; Pires et al. 2010). Despite these limitations, the included studies have a consistent and recurring message that oral piercings frequently cause hard and soft tissue complications. Thus, education of patients and increasing their awareness of these risks is imperative, and a key responsibility of healthcare professionals.

## Conclusion

The findings of the present systematic review evaluating the oral complications associated with metal ion release from oral piercings reveal the following:

- Oral piercings are associated with complications involving hard and soft dental tissues.
- Oral piercings leach metal ions into surrounding tissues which may cause local mucosal changes.
- Additionally, the evaluated studies showed that tongue piercings are commonly associated with tooth fractures, whilst lip piercings result in a greater incidence of gingival recession.

### Supplementary Information

Below is the link to the electronic supplementary material.

Supplementary file1 (DOCX 38 KB)

## Data Availability

Data and material the data used for this research can be made available upon request to the authors.
