# Supplementary material for: Oral complications associated with metal ion release from oral piercings: a systematic review
Source: Eur Arch Paediatr Dent. 2023 Aug 15;24(6):677–90. doi: 10.1007/s40368-023-00831-0 (PMC10657282; doi:10.1007/s40368-023-00831-0)
Supplement: Supplementary file 1 — Supplementary file1 (DOCX 38 KB) [file 40368_2023_831_MOESM1_ESM.docx]

**SUPPLEMENTAL DATA**

**Supplemental Data 1**. Search strategy applied according to each database.

| **Database** | **Search strategy (complications alone)** | **Number of Articles** | **Search strategy (metal ion release)** | **Number of Articles** |
| --- | --- | --- | --- | --- |
| **PUBMED**  <http://www.ncbi.nlm.nih.gov/sites/pubmed> | (Mouth [MESH] OR mouth*[tiab] OR oral[tiab] OR Lip[MESH] OR lip[tiab] OR cheek[tiab] OR buccal*[tiab] OR labial [tiab] OR Tongue[MESH] OR tongue[tiab] OR lingual*[tiab] OR frenulum[tiab] OR uvula[tiab] OR tooth*[tiab] OR gingiva[tiab] OR gums[tiab] OR mucosa[tiab]OR Papilla Interdental[Tiab] OR Philtrum [tiab])  AND  (piercing* OR Body Piercing[Mesh] OR Jewelry [MESH])  AND  (Complication* [tiab] OR Injury [tiab] OR Disease [tiab] OR Adverse reaction [tiab]) | 204 | (Mouth [MESH] OR mouth*[tiab] OR oral[tiab] OR Lip[MESH] OR lip[tiab] OR cheek[tiab] OR buccal*[tiab] OR labial [tiab] OR Tongue[MESH] OR tongue[tiab] OR lingual*[tiab] OR frenulum[tiab] OR uvula[tiab] OR tooth*[tiab] OR gingiva[tiab] OR gums[tiab] OR mucosa[tiab]OR Papilla Interdental[Tiab] OR Philtrum [tiab])  AND  (piercing* OR Body Piercing[Mesh] OR Jewelry [MESH])  AND  (Ions[Mesh] OR "Metals"[Mesh] OR metal*[tiab] OR ions[tiab] OR "titanium"[MeSH Terms] OR "titanium"[tiab] OR "steel"[tiab] OR "steel"[MeSH Terms] OR Alloys[Mesh])  AND  Complication* [tiab] OR Injury [tiab] OR Toxicity [tiab] OR Allergy [MESH] Allergy [tiab] OR Hypersensitivity [MESH] OR Hypersensitivity [tiab] OR Adverse reaction [tiab] | 27 |
| **SCOPUS**  <http://www.scopus.com> | TITLE-ABS-KEY (Mouth OR oral OR Lip OR cheek OR buccal* OR labial OR Tongue OR lingual* OR frenulum OR uvula OR tooth* OR gingiva OR gums OR mucosa OR Papilla Interdental OR Philtrum)  AND  TITLE-ABS-KEY (piercing* OR Body Piercing OR Jewelry)  AND  TITLE-ABS-KEY (Complication* OR Injury OR Disease OR Adverse reaction) | 434 | (Mouth OR oral OR Lip OR cheek OR buccal* OR labial OR Tongue OR lingual* OR frenulum OR uvula OR tooth* OR gingiva OR gums OR mucosa OR Papilla Interdental OR Philtrum)  AND  (piercing* OR Body Piercing OR Jewelry)  AND  (Ions OR "Metals" OR metal* OR "titanium" OR "steel" OR Alloys)  AND  (Complication OR Injury OR Toxicity OR Allergy OR Hypersensitivity OR Adverse reaction) | 12 |
| **WEB OF SCIENCE**  <https://www.webofknowledge.com> | (Mouth OR oral OR Lip OR cheek OR buccal* OR labial OR Tongue OR lingual* OR frenulum OR uvula OR tooth* OR gingiva OR gums OR mucosa OR Papilla Interdental OR Philtrum)  AND  (piercing* OR Body Piercing OR Jewelry)  AND  (Complication OR Injury Or Disease OR Adverse reaction) | 231 | (Mouth OR oral OR Lip OR cheek OR buccal* OR labial OR Tongue OR lingual* OR frenulum OR uvula OR tooth* OR gingiva OR gums OR mucosa OR Papilla Interdental OR Philtrum)  AND  (piercing* OR Body Piercing OR Jewelry)  AND  (Ions OR "Metals" OR metal* OR "titanium" OR "steel" OR Alloys)  AND  (Complication OR Injury OR Toxicity OR Allergy OR Hypersensitivity OR Adverse reaction) | 47 |
| **COCHRANE**  <https://www.cochranelibrary.com> | (Mouth OR oral OR Lip OR cheek OR buccal* OR labial OR Tongue OR lingual* OR frenulum OR uvula OR tooth* OR gingiva OR gums OR mucosa OR Papilla Interdental OR Philtrum) in Title Abstract Keyword AND (piercing* OR Body Piercing OR Jewelry) in Title Abstract Keyword AND AND (Complication OR Injury OR Disease OR Adverse reaction) in Title Abstract Keyword | 2 | (Mouth OR oral OR Lip OR cheek OR buccal* OR labial OR Tongue OR lingual* OR frenulum OR uvula OR tooth* OR gingiva OR gums OR mucosa OR Papilla Interdental OR Philtrum) in Title Abstract Keyword AND (piercing* OR Body Piercing OR Jewelry) in Title Abstract Keyword AND (Ions OR "Metals" OR metal* OR "titanium" OR "steel" OR Alloys) in Title Abstract Keyword AND (Complication OR Injury OR Toxicity OR Allergy OR Hypersensitivity OR Adverse reaction) in Title Abstract Keyword | 3 |
| **EMBASE**  <https://www.embase.com> | mouth:ti,ab,kw OR oral*:ti,ab,kw OR lip:ti,ab,kw OR cheek:ti,ab,kw OR buccal*:ti,ab,kw OR labial*:ti,ab,kw OR tongue:ti,ab,kw OR lingual*:ti,ab,kw OR frenulum:ti,ab,kw OR uvula:ti,ab,kw OR tooth:ti,ab,kw OR gingiva:ti,ab,kw OR gums:ti,ab,kw OR mucosa:ti,ab,kw OR 'interdental papilla':ti,ab,kw OR philtrum:ti,ab,kw AND piercing*:ti,ab,kw OR 'body piercing':ti,ab,kw OR jewelry:ti,ab,kw AND complication:ti,ab,kw OR injury:ti,ab,kw OR disease*:ti,ab,kw OR  'adverse event':ti,ab,kw | 132 | mouth:ti,ab,kw OR oral*:ti,ab,kw OR lip:ti,ab,kw OR cheek:ti,ab,kw OR buccal*:ti,ab,kw OR labial*:ti,ab,kw OR tongue:ti,ab,kw OR lingual*:ti,ab,kw OR frenulum:ti,ab,kw OR uvula:ti,ab,kw OR tooth:ti,ab,kw OR gingiva:ti,ab,kw OR gums:ti,ab,kw OR mucosa:ti,ab,kw OR 'interdental papilla':ti,ab,kw OR philtrum:ti,ab,kw AND piercing*:ti,ab,kw OR 'body piercing':ti,ab,kw OR jewelry:ti,ab,kw AND ions:ti,ab,kw OR 'metals':ti,ab,kw OR metal*:ti,ab,kw OR 'titanium':ti,ab,kw OR 'steel':ti,ab,kw OR alloys:ti,ab,kw AND complication:ti,ab,kw OR injury:ti,ab,kw OR toxicity*:ti,ab,kw OR allergy*:ti,ab,kw OR hypersensitivity:ti,ab,kw OR 'adverse event':ti,ab,kw | 30 |

**Supplemental data 2** Risk Bias assessment of included studies.

Risk of Bias summary author's judgments for each included study, assessed by the Joanna Briggs Institute (JBI). Critical appraisal checklist for Cross-sectional studies (a)

| **(a)** | Q1. Were the criteria for inclusion in the sample clearly defined? | Q2. Were the study subject and the setting described in detail? | Q3. Was the exposure measured in valid and reliable way? | Q4. Were objective, standard criteria used for measurement of the condition? | Q5. Were confounding factors identified? | Q6. Were strategies to deal with confounding stated? | Q7. Was the outcome measured in a valid and reliable way? | Q8. Was appropriate statistical anaylsis used? | Overall risk |
| --- | --- | --- | --- | --- | --- | --- | --- | --- | --- |
| Simoes et al. 2014 | 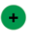 | 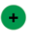 | 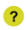 | 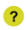 | 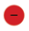 | 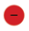 | 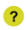 | 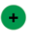 | High |
| Kapferer et al. 2012 | 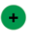 | 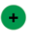 | 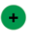 | 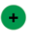 | 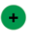 | 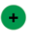 | 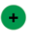 | 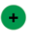 | Low |
| Vilchez-Perez et al. 2009 | 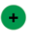 | 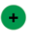 | 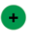 | 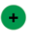 | 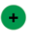 | 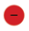 | 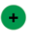 | 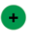 | Moderate |
| López-Jornet, et al. 2006 | 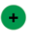 | 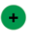 | 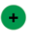 | 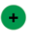 | 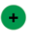 | 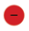 | 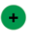 | 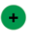 | Moderate |
| Campbell et al. 2002 | 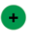 | 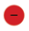 | 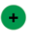 | 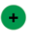 | 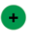 | 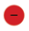 | 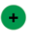 | 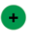 | Moderate |

Key:
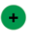
 Low risk
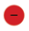
High risk
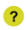
Unsure

Risk of Bias summary author's judgments for each included study, assessed by the Joanna Briggs Institute (JBI). Critical appraisal checklist for **case-control (b)**

| **(b)** | Q1. Were the groups comparable other than the presence of disease in cases or the absence of disease in controls? | Q2. Were cases and controls matched appropriately? | Q3. Were the same criteria used for identification of cases and controls? | Q4. Was exposure measured in a standard, valid and reliable way? | Q5. Was exposure measured in the same way for cases and controls? | Q6. Were confounding factors identified? | Q7. Were strategies to deal with confounding factors stated? | Q8. Was appropriate statistical analysis used? | Q9. Was the exposure period of interest long enough to be meaningful? | Q10. Was appropriate statistical analysis used? | Overall Risk |
| --- | --- | --- | --- | --- | --- | --- | --- | --- | --- | --- | --- |
| Tomazevic et al. 2017 | 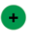 | 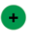 | 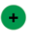 | 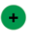 | 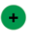 | 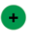 | 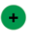 | 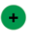 | 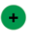 | 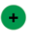 | Low |
| Ziebolz et al. 2012 | 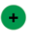 | 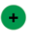 | 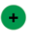 | 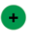 | 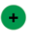 | 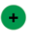 | 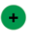 | 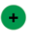 | 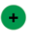 | 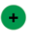 | Low |
| Lupi et al. 2010 | 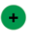 | 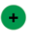 | 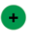 | 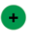 | 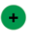 | 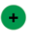 | 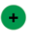 | 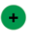 | 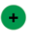 | 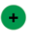 | Low |
| Pires et al. 2010 | 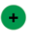 | 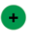 | 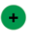 | 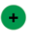 | 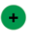 | 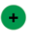 | 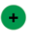 | 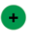 | 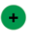 | 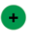 | Low |
| Kapferer et al. 2007 | 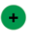 | 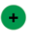 | 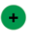 | 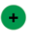 | 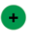 | 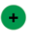 | 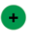 | 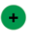 | 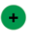 | 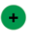 | Low |
| Dougherty et al. 2005 | 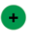 | 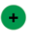 | 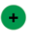 | 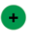 | 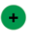 | 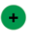 | 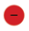 |  |  |  | Moderate |

Key: Low risk High risk Unsure

| **(c)**  Risk of Bias summary author's judgments for each included study, assessed by the Joanna Briggs Institute (JBI). Critical appraisal checklist for **cohort studies (c)** | 1.Were the groups similar and recruited from the same population? | 2.Were the exposures measured similarly? | 3. Was the exposure measured in a valid and reliable way? | 4. Were confounding factors identified? | 5. Were strategies to deal with confounding factors stated? | 6. Were the groups/participants free of the outcome at the start of the study (or at the moment of exposure)? | 7.Were the outcomes measured in a valid and reliable way? | 8. Was the follow up time reported and sufficient to be long enough for outcomes to occur? | 9. Was follow up complete, and if not, were the reasons to loss to follow up described and explored? | 10. Were strategies to address incomplete follow up utilized? | 11. Was appropriate statistical analysis used? | Overall risk |
| --- | --- | --- | --- | --- | --- | --- | --- | --- | --- | --- | --- | --- |
| Samoilenko et al. 2019 |  |  |  |  |  | N/A |  | NA | NA | NA |  | Low |
| Domingo et al. 2018 |  |  |  |  |  | N/A |  | N/A | N/A | N/A |  | Low. |
| Plessas et al. 2012 |  |  |  |  |  | N/A |  | N/A | NA | N/A |  | Low |
| Vieira et al. 2011 |  |  |  |  |  | N/A |  | NA | NA | NA |  | Moderate |
| Kapferer et al. 2010 | NA | NA | NA |  |  | NA |  | NA | NA | NA |  | Moderate |
| Oberholzer et al. 2010 |  |  |  |  |  | N/A |  | N/A | N/A | N/A |  | Moderate |
| Hickey et al. 2010 |  |  |  |  |  | N/A |  | N/A | N/A | N/A |  | High |
| Firoozmand et al. 2009 |  |  |  |  |  | N/A |  | N/A | N/A | N/A |  | Moderate |
| Slutzkey et al. 2008 |  |  |  |  |  | NA |  | NA | NA | NA |  | High |
| Ebrahim and Naidoo 2008 |  |  |  |  |  | N/A |  | N/A | N/A | N/A |  | High |
| López-Jornet, et al. 2006 |  |  |  |  |  | N/A |  | N/A | N/A | N/A |  | Moderate |
| Stead et al.2005 |  |  |  |  |  | N/A |  | NA | NA | NA |  | High |
| Levin et al. 2005 |  |  |  |  |  | N/A |  | NA | NA | NA |  | High |
| Kieser et al. 2005 |  |  |  | N |  | N/A |  | N/A | N/A | N/A |  | Moderate |
|  |  |  |  |  |  |  |  |  |  |  |  |  |

Key: Low risk High risk Unsure
